# Bilateral tuberculous psoas abscesses in an immunocompetent patient: a case report and review of the literature

**DOI:** 10.1099/acmi.0.001003.v4

**Published:** 2025-07-18

**Authors:** Hamid Laatiris, Hajar Zouaki, Yassine Benlahlou, Benaissa Elmostapha, Mariama Chadli

**Affiliations:** 1 Central Bacteriology Laboratory, Mohammed V Military Instruction Hospital, Rabat, Morocco; 2 Faculty of Medicine and Pharmacy, Mohamed V University of Rabat, Rabat, Morocco; 3 Rheumatology Department, Mohammed V Military Instruction Hospital, Rabat, Morocco

**Keywords:** immunocompetent, psoas abscess, tuberculosis

## Abstract

Psoas abscess is a rare infection historically associated with tuberculosis (TB), although non-tuberculous bacterial causes, particularly *Staphylococcus aureus,* have become increasingly common. This type of abscess can be either primary or secondary, and its diagnosis remains challenging due to the non-specific nature of clinical signs. Imaging and microbiological analyses are essential for establishing the diagnosis. We report the case of a 22-year-old patient with no significant medical history, who presented with persistent mechanical low back pain for 18 months. Initial computed tomography revealed a non-compressive disc protrusion, leading to treatment with non-steroidal anti-inflammatory drugs, without improvement. Further investigations revealed an extrapulmonary spinal localization of TB in an immunocompetent patient, with bilateral psoas abscesses caused by *Mycobacterium tuberculosis*, confirmed by the Ziehl–Neelsen staining, auramine staining, culture on Löwenstein–Jensen medium and GeneXpert PCR. Anti-TB treatment was initiated, resulting in favourable clinical evolution.

## Data Summary

No data was produced during this research, nor is any data necessary for reproducing the work.

## Introduction

Tuberculous psoas abscesses are rare and typically unilateral. Bilateral forms are even more uncommon and are mostly reported in immunocompromised patients. Historically linked to tuberculosis, this type of abscess is now undergoing an epidemiological shift, with an increasing incidence of non-tuberculous aetiologies, particularly pyogenic infections such as *Staphylococcus aureus* [1].

Psoas abscesses are classified as either primary, resulting from haematogenous spread from a distant infectious focus, or secondary, most often due to contiguous spread from spondylodiscitis or adjacent digestive tract infections [2]. The clinical presentation is often non-specific, commonly involving low back pain, intermittent fever and occasionally gait disturbances, which frequently delay diagnosis [3].

In endemic countries such as Morocco, tuberculosis remains a major cause of psoas abscesses, despite the increasing prevalence of pyogenic forms in industrialized nations. These extrapulmonary forms are often diagnosed late due to their atypical clinical presentation.

Diagnosis relies on imaging studies as well as microbiological investigations. Among the latter, the GeneXpert *Mycobacterium tuberculosis*/rifampicin (MTB/RIF) assay has emerged as a valuable diagnostic tool. It enables the rapid detection of *Mycobacterium tuberculosis* DNA within a few hours, with higher sensitivity than direct smear microscopy, especially in extrapulmonary specimens such as pus or biopsies. In addition, it identifies rifampicin resistance as a key marker of multidrug-resistant tuberculosis, thus facilitating appropriate therapeutic management.

We report a case of bilateral tuberculous psoas abscess in a young immunocompetent patient, managed in a tuberculosis-endemic setting. This case highlights the crucial role of molecular biology techniques in the early diagnosis of extrapulmonary tuberculosis.

## Case presentation

The patient was a 22-year-old man with no significant medical history, no history of smoking and no known risk factors such as diabetes, neoplastic disease, renal insufficiency or immunosuppressive therapy. He worked in manual labour involving heavy lifting and had no known contact with tuberculosis. He initially presented with mechanical-type low back pain (without nocturnal awakenings or morning stiffness and worsened by physical activity), evolving in an afebrile context. On clinical examination, the patient was stable and afebrile, with lumbar stiffness but no peripheral joint, enthesitic or extra-articular manifestations.

**Fig. 1.**
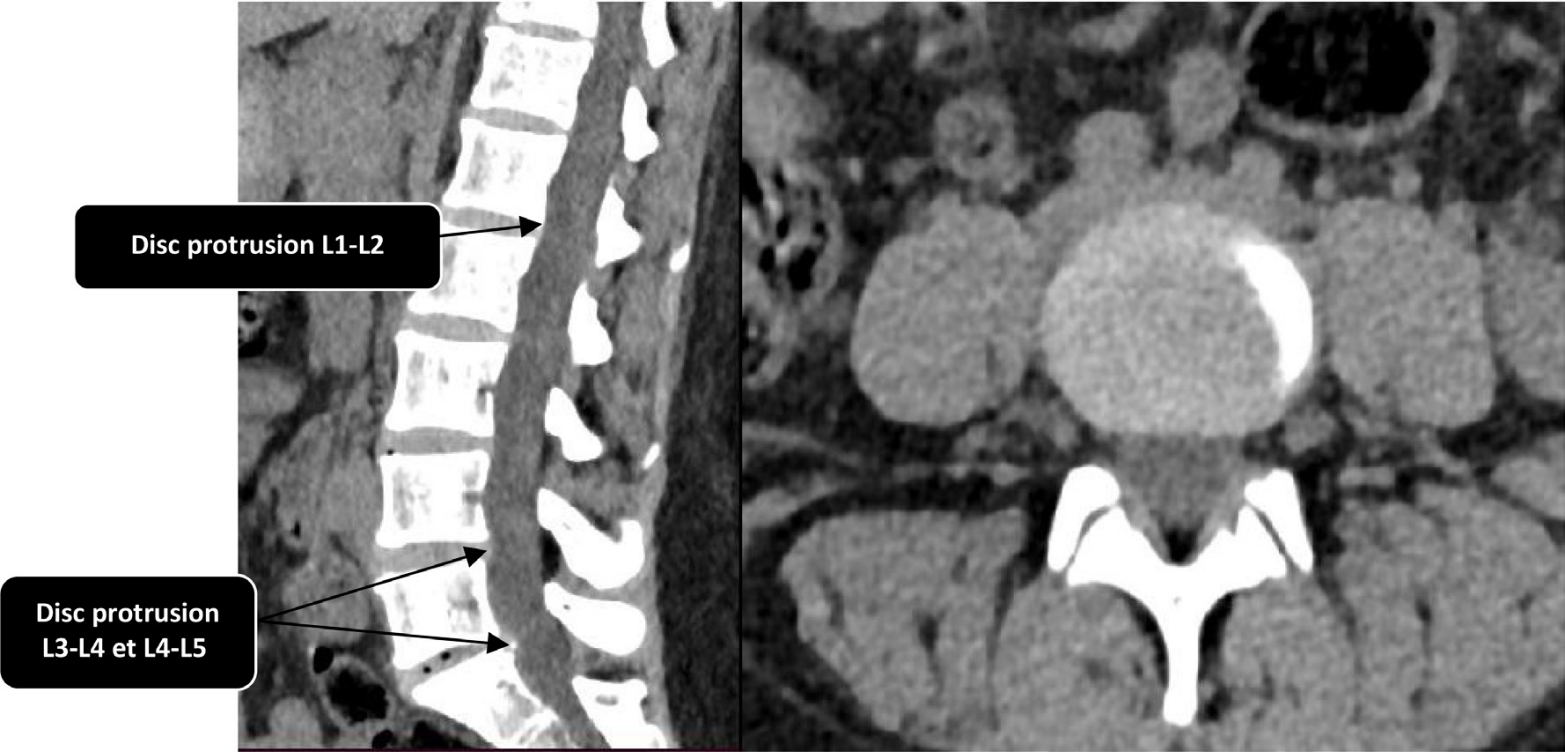
Lumbar CT scan in parenchymal window in axial section showing a harmonious global disc protrusion at the L1–L2, L3–L4 and L4–L5 levels, non-conflicting.

A lumbar computed tomography (CT) scan (Fig. 1) was requested by his general practitioner without prior medical analyses, given the mechanical nature of the pain. It revealed a non-compressive diffuse disc protrusion at levels L1–L2, L3–L4 and L4–L5. As a result, a 15-day course of 200 mg of celecoxib was prescribed. The patient was then lost to follow-up and self-medicated with analgesics (paracetamol) and various non-steroidal anti-inflammatory drugs (etoricoxib and indomethacin), with no clinical improvement.

After 18 months, the progression was marked by the persistence and worsening of his symptoms and the onset of bilateral pain in the lumbar fossae, which led to his consultation at the Rheumatology Department of Mohammed V Military Instruction Hospital, where a complete aetiological workup was performed.

Laboratory investigations revealed an inflammatory syndrome, with an elevated erythrocyte sedimentation rate (ESR) and a C-reactive protein (CRP) level of 82.4 mg l^−1^ (Table 1). Renal function was normal (creatinine within reference range), as were fasting blood glucose and body mass index, excluding malnutrition. Human immunodeficiency virus (HIV) serology was negative. The patient had received the Bacillus Calmette-Guérin (BCG) vaccination during childhood according to the national immunization schedule.

**Table 1.** Normal values for ESR and CRP

Parameter	Patient’s result	Reference value
ESR	80 mm	0–20 mm
CRP	82.4 mg l^−1^	0–5 mg l^−1^

A radiograph of the thoracolumbar spine revealed suspicious spinal abnormalities (lysis between L1/L2 and L2/L3).

**Fig. 2.**
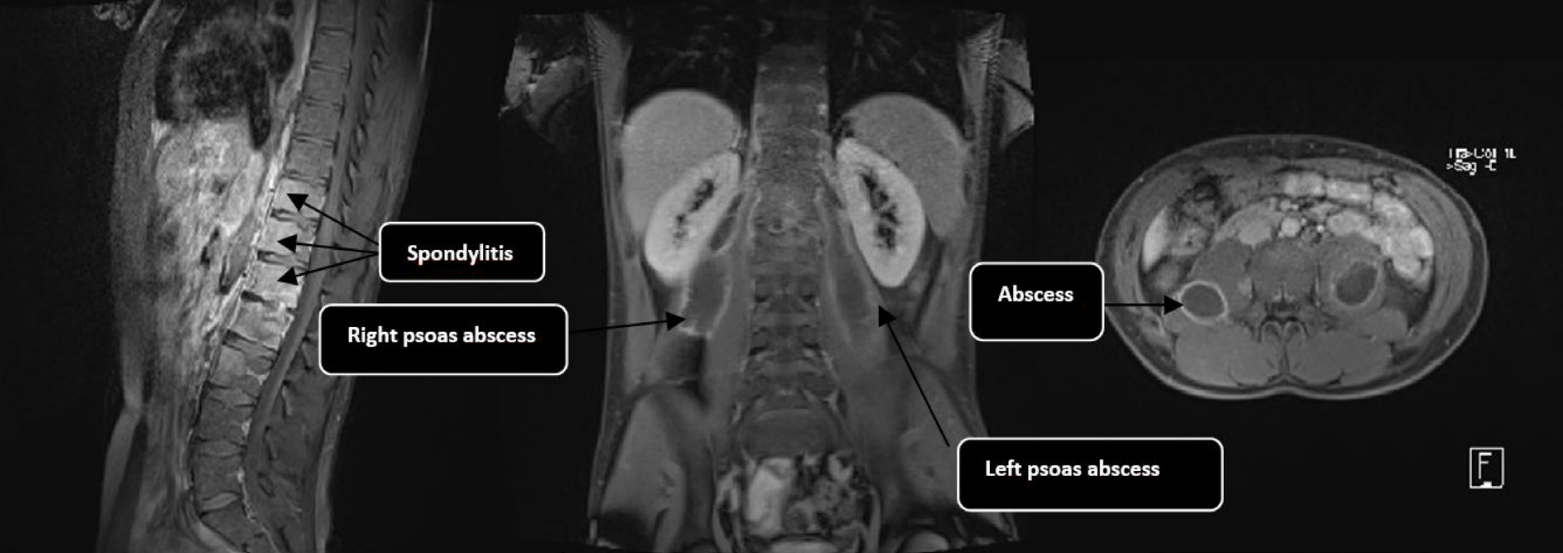
Evidence of infectious spondylodiscitis suggestive of tuberculosis, with an L1–L2 discal abscess extending into adjacent foraminal and paravertebral spaces and infiltrating the right and left psoas muscles.

Magnetic resonance imaging (MRI) revealed a discal abscess at the L1–L2 level, extending into the adjacent foraminal and paravertebral spaces, as well as into the right and left psoas muscles. These findings were secondary to tuberculous spondylodiscitis and not of primary origin. No abnormalities were observed in the pelvis or sacroiliac joints (Fig. 2).

In our case, the radiography could suggest other degenerative or neoplastic pathologies, but the MRI was decisive in identifying the infectious nature of the condition, ruling out rheumatic diseases such as spondyloarthritis due to the absence of sacroiliitis, specific spinal images and coxitis.

The main diagnostic dilemma in this case lies in the nature of the vertebral infection, as the patient lives in a tuberculosis-endemic country. The diagnosis of *M. tuberculosis* infection is not ruled out, even in the absence of signs of tuberculosis infection.

**Fig. 3.**
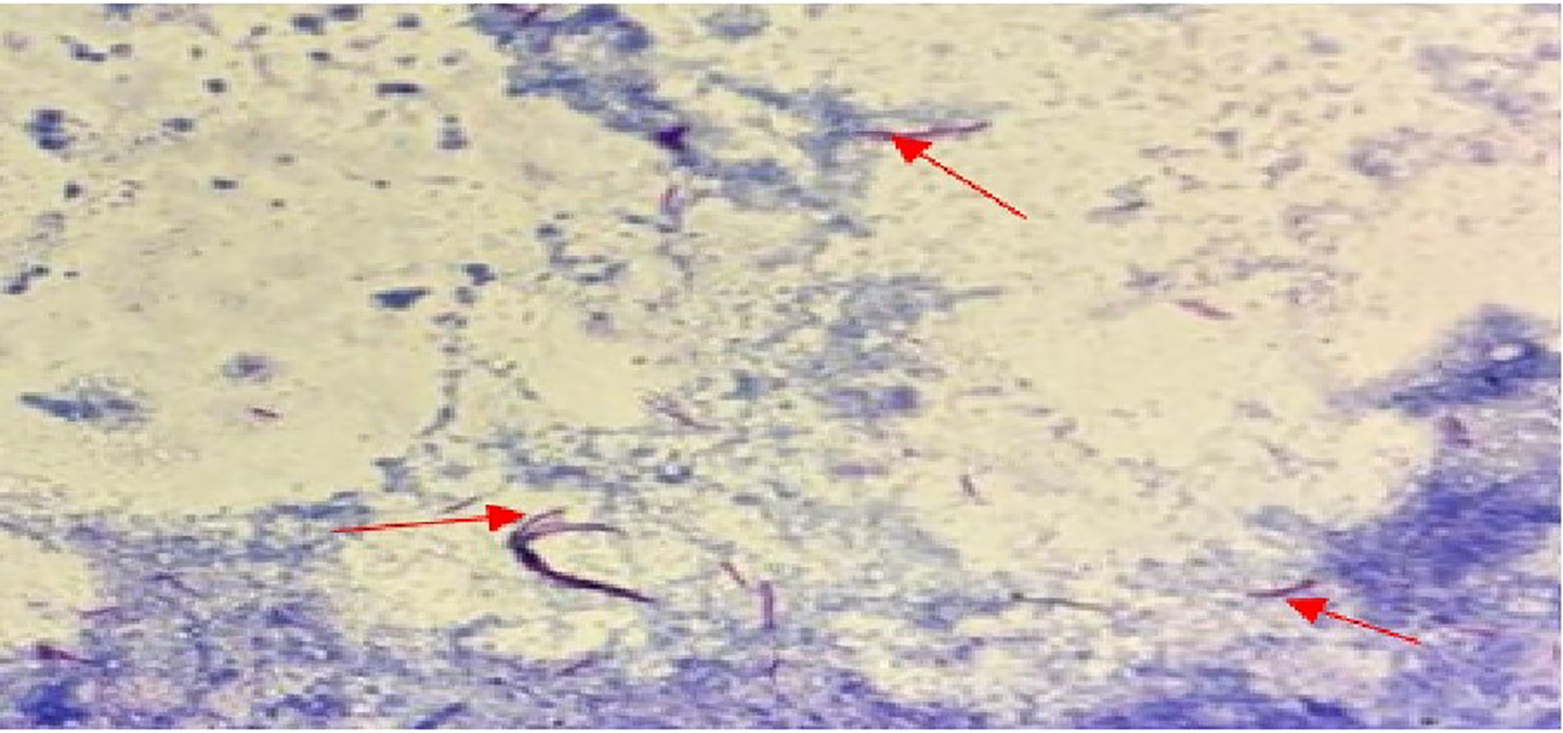
Presence of AFB from the *M. tuberculosis* complex in the Ziehl–Neelsen staining.

CT-guided drainage of the psoas abscesses was performed (Fig. 3). Analysis of the aspirated fluid revealed a marked cellular reaction, with initially sterile cultures on standard media. Direct examination with the Ziehl–Neelsen and auramine staining demonstrated the presence of acid-fast bacilli (AFB), suggestive of *M. tuberculosis* infection.

Culture was performed on solid Löwenstein–Jensen medium, incubated at 37 °C with weekly readings. Mycobacterial growth was observed on day 25, consistent with a paucibacillary form. Identification of *M. tuberculosis* was confirmed by the Ziehl–Neelsen staining and the GeneXpert MTB/RIF assay.

Molecular amplification using the GeneXpert MTB/RIF confirmed the presence of *M. tuberculosis* without any *rpoB* gene mutation, indicating no resistance to rifampicin. The bacterial load was reported as ‘*M. tuberculosis* detected high’. Phenotypic drug susceptibility testing for first-line anti-tuberculosis drugs was also performed on the isolated strain, showing no resistance to isoniazid, rifampicin, ethambutol or pyrazinamide.

Additionally, a standard chest X-ray was conducted to assess for concurrent pulmonary involvement. The findings were normal, with no signs suggestive of active tuberculosis.

As for sputum analysis, three consecutive samples were collected over 3 days. Each was tested by the Ziehl–Neelsen staining and GeneXpert MTB/RIF assay. All results were negative, effectively ruling out associated active pulmonary tuberculosis.

The patient was started on anti-tuberculosis treatment according to the national protocol recommended for extrapulmonary forms of tuberculosis. The regimen consisted of a 2-month intensive phase with four drugs: rifampicin (600 mg day^−1^), isoniazid (300 mg day^−1^), pyrazinamide (1,500 mg day^−1^) and ethambutol (1,200 mg day^−1^), followed by a 4-month continuation phase with rifampicin and isoniazid (2RHZE/4RH regimen).

This treatment was administered on an outpatient basis under strict follow-up as directly observed therapy, in accordance with the guidelines of the National Tuberculosis Control Program (PNLT). The patient showed favourable clinical and biological improvement.

A contact investigation was also carried out among individuals with close contact to the patient, particularly in his workplace. Screening included a clinical examination to identify potential respiratory symptoms and referral to a diagnostic centre for chest X-ray evaluation.

## Discussion

The interest of our case lies in the occurrence of a large bilateral psoas abscess in an immunocompetent, HIV-negative patient. Bilateral involvement is rare and typically observed in immunocompromised individuals. The literature contains few reports of bilateral psoas abscesses in immunocompetent patients. One such case described tuberculous spondylodiscitis complicated by bilateral abscesses in a young adult without immunosuppression [4].

Psoas abscesses are usually monomicrobial and can be classified as either primary or secondary. They account for ~5–10% of abdominal suppurations. Other isolated reports have described bilateral presentations in the absence of HIV infection, diabetes, malignancy or renal insufficiency. Our case falls within this rare category, highlighting the pivotal role of imaging and molecular testing in establishing the diagnosis, even in the absence of systemic symptoms or classical risk factors [5].

In immunocompetent patients, multifocal tuberculous psoas abscesses can manifest in a subacute manner with lower back pain, fever and sometimes hip movement limitations. These symptoms can be insidious and are often confused with other non-infectious pathologies, delaying the diagnosis. Potential complications include fistulization, extension to other anatomical regions or sepsis. MRI is the method of choice for diagnosing this condition, revealing intra-muscular fluid collections and sometimes involvement of adjacent structures, such as the vertebrae [6].

It is possible that pulmonary tuberculosis, as the initial focus, led to dissemination to other body sites. Indeed, tuberculosis generally spreads from the lungs to other parts of the body [7]. This dissemination occurs hematogenously, notably in spondylodiscitis, via vessels from the intercostal and lumbar vertebral arteries [8]. Thus, the sequence of involvement may be pulmonary, sternal, vertebral, psoas and finally femoral. The psoas muscle, located in an extra-peritoneal compartment, can be affected by pathological processes from supradiaphragmatic and intra- and retro-peritoneal regions, or even from the lower limbs [9]. The psoas abscess in this case is secondary to spondylodiscitis, as shown by several studies [10]. It is often associated with immunocompromised states such as HIV infection, malignant tumours, renal failure, intravenous drug abuse, diabetes mellitus or other chronic diseases and trauma [1]. However, our patient did not present these risk factors.

The patient’s HIV-negative status is relevant because HIV is known to facilitate the dissemination of *M. tuberculosis* to sites other than the lungs [4]. The clinical signs of psoas abscesses are nonspecific, often revealed by a prolonged infectious state accompanied by back pain, which may be febrile (pyogenic abscess) or non-febrile (cold abscess), and psoitis. As in our case, a marked biological inflammatory syndrome (elevated CRP, sedimentation rate and leukocytes) is frequently observed, but it is generally not useful for specific diagnosis [1].

The diagnosis of psoas abscess is primarily based on radiological examinations. CT scans are extremely sensitive (~100%), allowing assessment of abscess extent, detection of underlying lesions, guidance for microbiological analysis via puncture and orientation for drain placement for therapeutic purposes [11].

Cytobacteriological examination of the pus is crucial for identifying the pathogen and should preferably be done before starting antibiotic therapy or after a period of treatment cessation. *Staphylococci* are the most common pathogens in primary psoas abscesses (90% of cases), followed by *Streptococci* (5%) and *Escherichia coli* (3%) [12]. Less frequently involved pathogens in primary psoas abscesses include *Brucella* sp. and *M. tuberculosis* complex [13].

It is well established that the sensitivity of the Ziehl–Neelsen staining in extrapulmonary specimens is limited, particularly in paucibacillary forms. The false-negative rate can reach up to 60% in pus samples, which may significantly delay diagnosis if this method is used alone [14]. In comparison, culture on Löwenstein–Jensen medium remains the gold standard for the identification of *M. tuberculosis*, offering higher sensitivity but requiring an average incubation period of 3–6 weeks.

PCR, particularly via the GeneXpert MTB/RIF assay, provides a rapid alternative with a sensitivity exceeding 80% in purulent samples. The World Health Organization (WHO) recommends the systematic use of GeneXpert as the initial diagnostic test in cases of suspected extrapulmonary tuberculosis, including pus samples, due to its high efficacy and its ability to simultaneously detect rifampicin resistance [15]. In our case, rapid detection by GeneXpert enabled early and targeted therapeutic management.

The treatment of tuberculous psoas abscess relies mainly on prolonged anti-tuberculosis therapy (6–12 months), often supplemented by surgical or radiological drainage of the abscesses, depending on their size and initial response to treatment. In immunocompetent patients, the prognosis is generally favourable with early management, but delayed diagnosis can lead to severe complications, such as sepsis or extensive involvement [16].

## Conclusion

The tuberculous psoas abscess, although rare, remains a serious condition requiring appropriate management and should be considered when encountering any abscessed collection, especially in tuberculosis-endemic countries like ours. Radiology and microbiological techniques (PCR and specific cultures) played a key role in the diagnosis, and the management involved prolonged antibiotic therapy and appropriate surgical drainage. This case highlights the importance of a comprehensive diagnostic approach to identify tuberculous infections, even in the absence of obvious clinical signs.

## Abbreviations

AFB: acid-fast bacilli
BCG: Bacillus Calmette-Guérin
CRP: C-reactive protein
CT: computed tomography
ESR: erythrocyte sedimentation rate
HIV: human immunodeficiency virus
MRI: magnetic resonance imaging
MTB/RIF: *Mycobacterium tuberculosis*/rifampicin
TB: tuberculosis
